# Central Nervous System Involvement in ANCA-Associated Vasculitis: What Neurologists Need to Know

**DOI:** 10.3389/fneur.2018.01166

**Published:** 2019-01-10

**Authors:** Yang Zheng, Yinxi Zhang, Mengting Cai, Nanxi Lai, Zhong Chen, Meiping Ding

**Affiliations:** ^1^Department of Neurology, Second Affiliated Hospital, School of Medicine, Zhejiang University, Hangzhou, China; ^2^Department of Pharmacology, Key Laboratory of Medical Neurobiology of the Ministry of Health of China, College of Pharmaceutical Sciences, School of Medicine, Zhejiang University, Hangzhou, China

**Keywords:** anti-neutrophil cytoplasmic antibodies, vasculitis, granulomatosis with polyangiitis, microscopic polyangiitis, central nervous system

## Abstract

**Objective:** To provide a comprehensive review of the central nervous system (CNS) involvement in anti-neutrophil cytoplasmic antibody (ANCA)-associated vasculitis (AAV), including the pathogenesis, clinical manifestations, ancillary investigations, differential diagnosis, and treatment. Particular emphasis is placed on the clinical spectrum and diagnostic testing of AAV.

**Recent Findings:** AAV is a pauci-immune small-vessel vasculitis characterized by neutrophil-mediated vasculitis and granulomatousis. Hypertrophic pachymeninges is the most frequent CNS presentation. Cerebrovascular events, hypophysitis, posterior reversible encephalopathy syndrome (PRES) or isolated mass lesions may occur as well. Spinal cord is rarely involved. In addition, ear, nose and throat (ENT), kidney and lung involvement often accompany or precede the CNS manifestations. Positive ANCA testing is highly suggestive of the diagnosis, with each ANCA serotype representing different groups of AAV patients. Pathological evidence is the gold standard but not necessary. Once diagnosed, prompt initiation of induction therapy, including steroid and other immunosuppressants, can greatly mitigate the disease progression.

**Conclusions and Relevance:** Early recognition of AAV as the underlying cause for various CNS disorders is important for neurologists. Ancillary investigations especially the ANCA testing can provide useful information for diagnosis. Future studies are needed to better delineate the clinical spectrum of CNS involvement in AAV and the utility of ANCA serotype to classify those patients.

**Evidence Review:** We searched Pubmed for relevant case reports, case series, original research and reviews in English published between Sep 1st, 2001 and Sep 1st, 2018. The following search terms were used alone or in various combinations: “ANCA,” “proteinase 3/PR3-ANCA,” “myeloperoxidase/MPO-ANCA,” “ANCA-associated vasculitis,” “Wegener's granulomatosis,” “microscopic polyangiitis,” “Central nervous system,” “brain” and “spinal cord”. All articles identified were full-text papers.

## Introduction

Central nervous system (CNS) vasculitis, with its myriad and evolving presentations, always poses a great diagnostic challenge for neurologists. It occurs either as part of a systemic vasculitis, or a primary disorder restricted to the CNS (1). Anti-neutrophil cytoplasmic antibody (ANCA) -associated vasculitis (AAV), a systemic small-vessel vasculitis, is characterized by pathogenic ANCA production (1). In the clinical practice, AAV mainly includes granulomatosis with polyangiitis (GPA), microscopic polyangiitis (MPA) and eosinophilic granulomatosis with polyangiitis (EGPA) (1). Timely recognition and diagnosis of AAV is important, since the progressive disease can be dramatically mitigated by prompt use with steroid and other immunosuppressive agents.

Neurologic involvement is not uncommon in AAV throughout the disease course, ranging from 22 to 54% in patients with GPA (2–5) and 34 to 72% in those with MPA (2–4). CNS is affected in < 15% of patients with AAV (5) but accounts for much of the morbidity in those patients (1–3, 7–10). However, the heterogeneous CNS symptoms in AAV may hinder early diagnosis among neurologists, causing treatment delays and disease progression, leading to relapses, or even death.

Therefore, this review aims to increase the awareness of AAV among neurologists. We mainly focus on GPA and MPA, which have distinctive features compared with EGPA (6–10). This review comprehensively illustrates the pathogenesis, CNS manifestations, ancillary investigations, and treatment algorithms warranted for AAV patients with CNS involvement. In particular, we put a special emphasis on its clinical spectrum and the utility of ACNA testing in diagnosing and subtyping those patients.

## Pathophysiology

**Figure 1:**
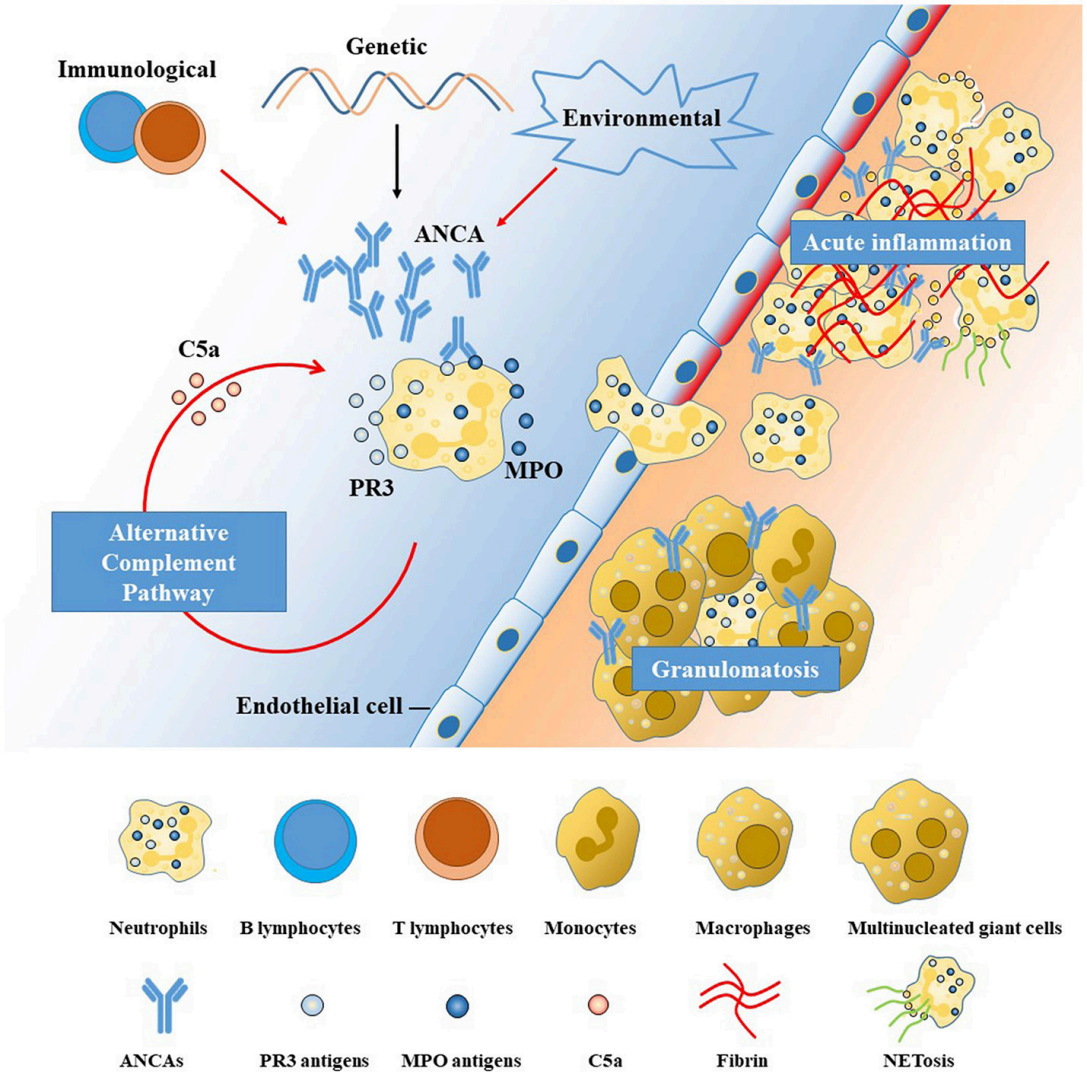
Pathogenesis of anti-neutrophil cytoplasmic antibody (ANCA)-associated vasculitides (AAV). The left side of the diagram (with blue background) represents the blood stream and the right (with orange background) the interstitial tissue, separated by a line of endothelial cells. ANCAs are autoantibodies directed against proteins in the cytoplasmic granules of neutrophils. The two antigenic targets are proteinase 3 (PR3) and myeloperoxidase (MPO) normally expressed on the surface or inside the cytoplasm of resting neutrophils (11, 12). The interplay among genetic, environmental, and immunological factors contributes to the high membrane expression and release of PR3 and MPO, leading to the production and proliferation of pathogenic ANCAs. Primed neutrophils are activated by ANCAs and transmigrate the vessel wall, undergoing respiratory bursts, degranulation, and neutrophil extracellular traps (NETs) generation (11), which are further augmented by the alternative complement pathway (13). The neutrophil-mediated processes are the major contributor to the injury and inflammation of the endothelial cells lining the vascular wall in the early phase (14). Monocytes are subsequently recruited at sites of acute inflammation and necrosis, inducing the development of granulomatous inflammation mainly mediated by an exaggerated monocyte/macrophage reaction (11). Potential treatment targets are illustrated by red arrows in the figure, including the T-cell and B-cell dysregulation, environmental triggers (microbes, drugs), aberrant activation of alternative complement pathway and NETs. ANCA, anti-neutrophil cytoplasmic antibody; MPO, myeloperoxidase; PR3, proteinase 3; NET, neutrophil extracellular trap.

A basic understanding of how pathogenic ANCAs are induced, take effect and invade the CNS helps to understand its manifestations and illuminate potential targets for treatment in AAV. Pathogenic ANCAs, targeting mainly at proteinase 3 (PR3) and myeloperoxidase (MPO) expressed by innate immune cells, are the major contributor to the pathogenesis of AAV, according to *in vitro* and *in vivo* experimental data (11). An overview of the pathophysiology is shown in Figure 1. Pathogenic ANCAs are induced by the interplay of multiple environmental, genetic, and immunological factors (8, 11, 15). An encounter with the antisense peptides of PR3 or MPO triggers the immunological self-amplication network (11). The antigen-recognition capability of each individual, however, is more likely genetically determined (8). In addition, the generation of pathogenic ANCAs is further facilitated by an impaired immunological regulation, as in the pathogenesis of the few treatable neurological disorders (16–19). The function of regulatory T (T_REG_) cells and regulatory B cells with CD5 expression are suppressed, whereas the circulating effector memory T cells (T_EM_) (20) and ANCA-producing B cells (15) are proliferated and activated.

Following the generation of pathogenic ANCAs, different pathways lead to the two major pathological changes in AAV, namely vasculitis, and granulomatosis. Neutrophils, activated by pathogenic ANCAs and fueled by the alternative complement pathway (13), play the central role. Activated neutrophils can transmigrate the vessel wall and undergo respiratory burst, degranulation, neutrophil extracellular traps (NETs), apoptosis and necrosis (14), causing disruptions of the endothelium and thus activation of the coagulation cascade, leading to fibroid necrosis at sites of vasculitic inflammation. This neutrophil-activation process is further augmented by the complement system, especially the alternative pathway, with C5a playing a key role in-between (13). By contrast, the pathogenesis of extravascular granulomatosis is less well-understood. Current thinking holds that the chronic inflammation is initiated by the acute neutrophil-mediated necrosis (21). Subsequently, defects in the cell death machinery and aberrant reaction of monocytes and macrophages contribute to the chronic inflammation and granulomatosis formation in AAV.

CNS can be affected in AAV through one of the following pathways (22, 23): (1) inflammation, obstruction or increased permeability of the small to medium-sized cerebral vessels due to systemic vasculitis; (2) infiltration or compression of granulomatous pathology from adjacent structures; (3) granulomatous lesions developing *de novo* within the CNS. Mechanisms vary according to the specific CNS structures involved. In general, extra-axial lesions involving the dura, or pituitary gland are mainly attributed to granulomatous inflammation, while parenchyma pathologies are mediated by vasculitis and breakdown of blood brain barrier (24). However, it remains unclear whether pathogenic ANCAs are produced intrathecally or from the systemic circulation and how the two ANCA serotypes contribute to different CNS manifestations.

## The Many Faces of AAV With CNS Involvement

### Epidemiology

AAV, especially GPA and MPA, is a multisystem disease. Up to 40–50% of patients have a remitting-relapsing course (7, 12). The onset of CNS flare is mostly acute or subacute, depending on the specific neurological syndrome. CNS symptoms usually present late in the disease course (5, 25). No gender predilection is observed and most patients tend to have their first CNS flare in the middle age (5, 25, 26).

### Overview of Systemic AAV

It is of great help to know the accompanying systemic symptoms at the time of or prior to CNS flare. A long-term history of constitutional symptoms like fever, weight loss, fatigue and arthralgia are often suggestive of an autoimmune etiology. In addition, organs susceptible to AAV damage include ear, nose and throat (ENT), lung and kidney (6). ENT involvement for over 3 months, presenting as chronic sinusitis, otitis media, or mastoiditis, is diagnostic of AAV, (3, 7, 24). History of renal dysfunction (proteinuria, hematuria and acute kidney failure) and pulmonary problems (pulmonary nodules, infiltration, alveolar hemorrhage, chronic cough, asthma, or rarely respiratory failure) were also frequently reported. Therefore, for patients suspected with AAV, an investigation of systemic symptoms is warranted. Manifestations highly suggestive of AAV are listed in Table 1.

**Table 1:** Systemic manifestations of AAV other than CNS^#^.

**Involved organ**	**Manifestations**
Constitutional symptoms	Fever, weight loss, polyarthralgia, polymyalgia, malaise, polyarthritis
Ear nose and throat (ENT)	Chronic sinusitis^*^, chronic otitis media^*^, chronic mastoiditis^*^, bloody nasal discharge/crusts/ulcers/granuloma^*^, subglottic stenosis^*^
Trachea and lung	Hemoptysis (due to pulmonary hemorrhage^*^ or tracheobronchial disease), lung nodules^*^, cough, dyspnea, pleuritic pain
Kidney	Glomerulonephritis (especially rapidly progressive glomerulonephritis)^*^
Skin	Leukocytoclastic angiitis^*^ (typically palpable purpura involving the lower extremities with focal necrosis and ulceration)
Eye and orbit	Epislceritis/scleritis^*^, uveitis, conjunctivitis, corneal ulceration, retinal vasculitis, optic neuropathy, retro-orbital mass or inflammation^*^
Peripheral nervous system	Mononeuritis multiplex^*^, cranial neuropathies^*^
Gastrointestional tract	Diarrhea, nausea, vomiting, abdominal pain, gastrointestinal hemorrhage; Elevated liver enzymes
Cardiovascular	Ischemic cardiac pain, cardiomyopathy, congestive heart failure, loss of pulses, valvular heart disease, pericarditis

# *The table is adapted from previous reviews on related topics (6, 12, 27)*.

* *Clinical features highly suggestive of AAV (6, 28)*.

*AAV, anti-neutrophil cytoplasmic antibody-associated vasculitis; CNS, central nervous system*.

### Clinical and Imaging Spectrum of CNS Involvement in AAV

CNS presentations in AAV patients vary, including headache, ischemic infarction, intracranial hemorrhage, encephalopathy (seizures, neuropsychiatric disorders, confusion, or altered consciousness), and rarely spinal cord symptoms. Those symptoms are caused by the involvement of corresponding CNS structures including the dura mater, brain parenchyma, pituitary gland, spinal cord, and leptomeninges with decreasing frequency.

#### Brain Parenchyma Involvement

**(a)Cerebrovascular events**
  Ischemic infarctions and intracranial hemorrhages, though rare, can be the initial presentation of AAV and are always associated with significant morbidity (29– 31). Timely recognition of AAV as the underlying cause is difficult at the first visit in the emergency room due to its rarity. Therefore, the distinguishing features of AAV-related strokes can aid in early diagnosis and improve prognosis. Ischemic infarctions typically present as an isolated or multiple lesions affecting the white matter, since distal penetrating vessels are predominantly affected. Medullary and pontine infarctions were also reported in some cases (29, 30). Ischemic infarctions caused by AAV are typically resistant to antiplatelet therapy and tend to recur without proper immunosuppressive therapy. Patients with AAV are also at an increased risk of hemorrhagic transformation after reperfusion therapy of ischemic stroke (32). Hemorrhagic events occur less often. They more often affect the brain parenchyma (31), and sometimes the subarachnoid space (33). Brain Imaging findings usually correspond to the specific disease in each patient, involving ischemic, hemorrhagic lesions, or variable degrees of small-vessel diseases affecting both white and gray matter (34). Nonspecific white matter lesions with T2 hyperintensities can appear in the periventricular, subcortical regions, the basal ganglia, the mesencephalon and pons.
**(b)Posterior reversible encephalopathy syndrome**
  Posterior reversible encephalopathy syndrome (PRES) is a rare yet unique complication in the late phase of AAV (35, 36). Clinically, this entity typically presents with an acute onset. Symptoms are generalized and include encephalopathy, seizures, headache and visual disturbance. Brain imaging typically reveals findings consistent with vasogenic edema, predominantly involving the bilateral parieto-occipital regions. Most patients with PRES have a dramatic improvement within days to weeks, with only the supportive therapy. Symptoms may recur when the underlying AAV is not well-controlled.
**(c)Isolated parenchymal mass lesions**
  Isolated parenchymal mass lesions were very rarely reported in AAV (37–39), which often present with a discrete granuloma. Symptoms vary depending on the location of lesions, with seizures as the most frequent presentation (38). Brain magnetic resonance imaging (MRI) of isolated parenchymal granulomas reveals a well-delineated mass with a high signal intensity on T2-weighted images and enhancement on gadolinium-enhanced sequences (39).
**(d)Cognitive impairment**
  Cognitive decline, though mostly subclinical and mild, can occur in AAV patients as well, with an estimated prevalence of 30% (40). According to one study of 13 AAV patients (40), the pattern of cognitive impairment, mainly affecting abstract reasoning, attention and non-verbal memory, is different from that of age-related dementia. On brain MRI, multiple white matter lesions, mainly located in the periventricular or juxtacortical areas, are often associated with the cognitive impairment (40).

#### Brain Meninges Involvement

Pachymeninges is affected more frequently than leptomeninges (41). The frequency of hypertrophic pachymeningitis (HP) in adult AAV ranges from 18 to 35%, depending on the methodology of studies and the sample populations selected (41, 42). Manifestations of AAV-related pachymeningitis vary depending on the location and extent of inflammation. Headache, the dominant symptom of HP, is often severe and resistant to analgesics (22). Besides, neck stiffness is not commonly seen along with headache. Cranial nerves may be compressed by the thickened dura mater and cause symptoms such as visual loss, double vision, and facial palsies. Other times the pachymeningeal inflammation may infiltrate the brain parenchyma and cause impaired consciousness and seizures (24, 42). Notably, an entity named “CNS-limited AAV” was recently proposed by Yoloseki et al. for patients with MPO-ANCA-positive hypertrophic pachymeningitis, characterized by an elderly female predominance, less severe neurological damages, and lower rates of developing into the generalized disease (24). Yet it remains unclear whether the “CNS-limited AAV” represents a novel AAV phenotype or merely a transient disease stage (24).

Imaging studies are valuable in identifying hypertrophic thickening of the dura mater, monitoring disease activity and assessing the damage of adjacent structures. On brain MRI and computed tomography (CT) scans, HP typically shows linear thickening of dura mater or a bulging mass with enhancement. Gadolinium-enhanced T1-weighted MR imaging offers certain advantages over other sequences in identifying active inflammation of the thickened dura mater with superior spatial resolution. The brain pachymeningitis can involve the tentorium cerebelli, cranial fossa, cavernous sinus, falx cerebri, or convexity with no site preference. Additionally, brain-imaging studies can help assess the potential sinonasal and orbital involvement, a common occurrence in AAV. Additionally, the damage of adjacent bone structures is prominent on brain CT scans (41, 43).

#### Pituitary Gland and Stalk Involvement

Inflammation of the pituitary, termed hypophysitis, is rarely seen in AAV, yet requiring special consideration (44–46). Constitutional symptoms including fatigue, lethargy, headache, weight loss, and appetite loss frequently occur. Endocrine disturbances most commonly include diabetes insipidus, and hypogonadism (45). Other less common ones include hypothyroidism, adrenocorticotropic hormone (ACTH) deficiency and growth hormone (GH) deficiency (45, 47). Pituitary stalk compression resulting from pituitary enlargement may cause hyperprolectinemia as well (22). Visual deficits result with optic chiasm compression (45). Outcome of hypopituitarism is less favorable, despite the treatment with immunosuppressive agents. Pituitary dysfunction tends to persist, even though other systemic symptoms can come to remission after standard treatment (45, 46).

Brain MRI typically shows an enlarged pituitary gland or thickened stalk with peripheral enhancement on post-contrast sequences. Other findings include the lack of posterior pituitary hyperintensity on T1 images. Normal MRI, as reported in some cases, does not exclude pituitary involvement (47).

#### Spinal Cord Involvement

In general, spinal cord is rarely involved in AAV (48). The three possible mechanisms underlying spinal cord involvement include necrotizing inflammation of the spinal vasculature, compression of the spinal cord by inflamed thickened meninges as well as the formation of primary spinal granulomas (48). Clinical syndromes of the spinal cord include hypertrophic pachymeningitis (41, 48) and compressive myelopathy (49). Contrast-enhanced MRI of the spinal cord is of great diagnostic value and biopsy is often warranted to confirm the diagnosis.

#### Non-CNS Entities Highly Associated With CNS Involvement

**(a)Cranial neuropathy**
  Cranial neuropathies are rarely the only manifestation in AAV, but rather coexist with other CNS symptoms such as headache and systemic symptoms. Previous investigations found a prevalence between 2 and 10% of cranial neuropathies in patients diagnosed with GPA (50) and MPA (51, 52). Commonly affected cranial nerves in AAV include cranial nerve II–VIII (50). Bulbar palsy, by contrast, is less commonly involved (53).
**(b)Orbital disease**
  Eye involvement was reported in around 30–50% of patients diagnosed with GPA (54), with the orbit and sclera most frequently involved. MPA, by contrast, rarely impair eye movement (52). Symptoms vary and typically include proptosis, diplopia, decreased vision and orbital pain (55), resulting from compressions of ocular granuloma, granulomatous inflammation of the optic nerve and ischemic optic neuropathy due to vascular occlusion (56). MR imaging and CT can help reveal mucosal and bony lesions in the orbit and rule out continuous extension.
**(c)Parasinal disease**
  For patients with CNS manifestations suspected of AAV, an evaluation of paranasal sinuses is a must. Findings indicative of granulomatosis in the paranasal sinuses include a soft-tissue mass, thickening of the sinus wall, or “ground-glass” material in the lumen (34). As in orbital involvement, non-contrast CT scan has its diagnostic value in evaluating the nasal cavity, paranasal sinuses, mastoids and temporal bone, due to a better resolution of mucosal and bony pathologies compared with MRI (43). Brain MRI may also aid in the evaluation of soft tissue masses within those cavities.

#### Differences Between ANCA Serotypes

There is an increasing consensus that the ANCA serotype, either MPO-ANCA or PR3-ANCA, has a crucial role in AAV (12). Distinctively, previous studies revealed that the ANCA serotype represents two separate groups of patients in epidemiology, genetic background, clinical features, laboratory features as well as prognosis. We summarized the differences between MPO- and PR3-ANCA positive AAV in Table 2. Specifically, for AAV patients with CNS involvement, the ANCA serotype matters as well (24). Though the two serotypes tended to affect the nervous system with a similar frequency (57), they differ in the pattern and severity of CNS involvement. According to one study of patients with HP in Japan, MPO-ANCA-positive HP had an elderly female predominance, more frequently had lesions limited to the dura mater and upper airways, and was less likely to have a generalized systemic progression. On the contrary, PR3-ANCA-positive HP tended to have more severe neurological damages and a generalized disease progression (24). However, evidence remains limited to patients with HP. Extrapolation of the utility of ANCA serotype in other CNS manifestations remains to be further studied.

**Table 2:** Comparison of PR3- and MPO-ANCA-positive AAV.

**Features**	**PR3-ANCA-positive AAV**	**MPO-ANCA-positive AAV**
Pathophysiology 12, 57	Apoptosis of endothelial cells; Release of sFlt1 by monocytes; No established mouse model	Production of intracellular oxidants; No induction of release of sFlt1 by monocytes;Pathogenicity of autoantibody proved in mouse models
Epidemiology 12, 57	Northern Europe, America and Australia	Southern Europe and Asia
Genetic background 8, 12, 57	HLA-DP, SERPINA1, PRTN3	HLA-DQ, CTLA4
Clinical features 12, 57–59	More ENT involvement;Cavititating pulmonary lesions, nodules and masses; More organs involved	More often renal-limited;Fibrosing pulmonary lesions and patchy infiltrates
Pathological features 12	Granuloma and vasculitis	Granuloma and fibrosis
Response to induction therapy 12, 60	Better response to rituximab than cyclophosphamide	Increased risk of initial treatment failure
Long-term outcome 12	More relapsesBetter prognosis	Less relapsesHigher risk of long-term kidney and alveolar damageWorse prognosis

*PR3, proteinase 3; MPO, myeloperoxidase; ANCA, anti-neutrophil cytoplasmic antibodies; AAV, anti-neutrophil cytoplasmic antibody-associated vasculitis; HLA, human leukocyte antigen; ENT, ear, nose and throat*.

## Diagnosis

The diagnosis of CNS involvement in AAV, similar to the disease overall, requires consideration of clinical, serological, radiographic, and, when available, pathological evidence (61). In patients with established AAV, new-onset neurologic deficits with abnormal radiological and cerebrospinal fluid (CSF) findings are suggestive of CNS involvement. For patients without established underlying disease, neurologic symptoms closely compatible with CNS syndromes known to arise in AAV warrant further investigations for the systemic disease. In either case, ancillary tests are of important value in making the diagnosis and excluding other causes of the CNS abnormalities (Box 1). This section describes workup that can aid in the diagnosis of AAV with CNS involvement, and with special interest, pays extra attention to the value of ANCA testing in-between.

**Box 1:** Investigations in AAV-related CNS involvement.

ANCA immunoassays (MPO- and PR3- ANCA testing)
CBC, CMP, CRP, ESR (to evaluate the organ functions and the state of
inflammation)
Autoimmune panel, complement levels (to differentiate from other inflammatory
conditions)
Endocrine panel (to evaluate the pituitary function)
Screening for HIV, hepatitis, and tuberculosis
Analysis of cerebrospinal fluid (measure inflammatory mediators and
degradation proteins, assess the blood-brain barrier and exclude infection)
Chest CT
Brain imaging (CT; MRI, suggested sequences include T1/T2 weighted-
imaging, FLAIR, DWI, SWI and contrast enhanced T1 sequences)
Biopsy
AAV, anti-neutrophil cytoplasmic antibody-associated vasculitis; ANCA, anti-neutrophil cytoplasmic antibodies; MPO, myeloperoxidase; PR3, proteinase 3; CBC, complete blood count; CMP, comprehensive metabolic panel; CRP, C reactive protein; ESR, erythrocyte sedimentation rate; HIV, human immunodeficiency virus; CT, computed tomography; MRI, magnetic resonance imaging; FLAIR, fluid-attenuating inversion recovery; DWI, diffusion-weighted imaging; SWI, susceptibility-weighted imaging.

### ANCA Serology

ANCA testing is strongly recommended for patients with clinical features suggestive of AAV (Table 1) (6, 62). Its value lies mainly in AAV screening, and when positive, warrants further investigations for confirmative diagnosis. Positivity of the test is highly suggestive of AAV but not diagnostic by itself (6). However, the combination of ANCA positivity and certain clinical features are sufficient for AAV diagnosis, according to the widely-used classification algorithm proposed by Watts (28). A negative ANCA immunoassay, which occurs in up to half of pathologically diagnosed GPA and MPA, does not exclude AAV (6, 63). The negativity may be related to the limited sensitivity of ANCA testing, especially at the early stage of disease without systemic involvement (7). The diagnosis of AAV cannot be excluded merely based on a negative ANCA test. Therefore, biopsy of the affected organ is required for seronegative patients (6).

Regarding the testing techniques of ANCA, the most recent consensus recommended high-quality antigen-specific immunoassays for MPO- and PR3- ANCAs detection (6). ELISAs are the preferred screening methodology with a high sensitivity and specificity. Indirect immunofluorescence (IIF) for cytoplasmic ANCA (C-ANCA) and perinuclear ANCA (P-ANCA) detection is no longer prioritized as the screening test, given its large variability and poor diagnostic accuracy (6). When necessary, performing another assay or testing with another different methodology can yield higher sensitivity and specificity (6). In cases of emergency such as pulmonary-renal syndrome, rapid screening assays for ANCAs, including dot blots and biochip technology, are also available (64).

Controversy remains in the role of ANCA levels in monitoring disease activity and prediction of prognosis (63). Published studies reveal inconsistent results regarding the role of ANCA levels in predicting clinical relapse and reflecting disease activity (65–67). A meta-analysis in 2012 concluded that both a rise in ANCA and persistently positive ANCA were strongly associated with disease relapse and had a modest predictive value (66). Furthermore, results from a recent single-center study indicated that the predictive value of ANCA was only significant in renal-involved AAV, compared with those without renal involvement (68). Overall, current thinking holds that ANCA levels alone are helpful but not sufficient to determine relapse or reflect disease activity (6), and ANCA testing is not recommended to guide clinical decisions on treatment (61). Nevertheless, severe relapses are unlikely without elevated ANCA levels (69). The significance of serial monitoring remains to be proven.

### Other Laboratory Investigations

A diligent workup should be ordered for patients suspected of AAV, including complete blood count (CBC), complete metabolic profile (CMP), acute phase reactants, the autoimmune panel, the endocrine panel, the complement level and investigations for potential infections. For patients with endocrine dysfunction or abnormal pituitary on MRI, levels of pituitary hormones also need to be investigated. Laboratory tests typically show elevated markers of inflammation. Leukocytosis, thrombocytosis, normochromic normocytic anemia, elevated erythrocyte sedimentation rate (ESR) and C-reactive protein (CRP) values are indicative of the diagnosis (3, 7). Kidney functions, including serum creatinine and urinalysis, are warranted for the evaluation of renal injury. Regarding the autoimmune panel, autoantibodies other than ANCAs can be present (63), including antinuclear antibodies, rheumatoid factors, IgG4, anti-glomerular basement membrane antibodies and antiphospholipid antibodies, with unknown significance (63). Lastly, for differential diagnosis, investigations for potential infections (tuberculosis, human immunodeficiency virus, hepatitis) and complement levels are also required.

### Cerebrospinal Fluid

Lumbar puncture is warranted for suspected patients as well. CSF analysis has a high sensitivity but low specificity in the diagnosis of AAV-related CNS disease. Most had mild lymphocyte-predominant pleocytosis, elevated protein levels and normal glucose level (7, 25, 37). Therefore, a normal CSF analysis makes the diagnosis of AAV less likely. Infectious etiology and flow cytometry for atypical cancer cells should be tailored to clinical manifestations and risk factors.

### Pathology

Histopathology is the gold standard for diagnosis of small vessel vasculitis. Samples can be taken from affected organs, most commonly the kidney and the skin. Lung and nasal biopsies are rarely performed, limited by the high rate of false negatives. Brain biopsies can also be taken from corresponding structures including the dura, brain parenchyma and the overlying leptomeninges. Typically two types of pathological findings have been described: (1) necrotizing vasculitis affecting small to medium vessels; (2) granulomatosis with inflammatory cell infiltration (monocytes, plasma cells, eosinophils, and polymorphonuclear leukocytes) (41). Fibrinoid necrosis and edema were also detected in some cases. In patients of HP, fibrosis of the dura mater was always present as well (24, 41). A negative yield of biopsy, however, does not exclude the diagnosis of AAV due to its segmental nature of lesions.

### Differential Diagnosis

AAV must be distinguished from ANCA-positive conditions where ANCA does not exert a direct role in pathophysiology (Box 2). Multiple conditions other than AAV show an elevated titer of serum ANCAs. Nonetheless, ANCAs do not mediate a direct pathogenic role, but rather indicate a chronic immune response of neutrophil cell death most times (63). Compared with the mimics, a high level and affinity of ANCAs and clinical features suggestive of AAV are important clues for the correct diagnosis (6).

**Box 2:** Conditions other than AAV with positive ANCA immunoassays^#^.

**Vasculitis of other causes**
Anti-GBM disease, IgA-vasculitis^*^
**Gastrointestinal diseases**
Ulcerative colitis, Primary Sclerosing Cholangitis, Inflammatory liver disease
**Systemic inflammatory conditions**
IgG4-related disease, Rheumatoid arthritis, Systemic lupus erythematous
**Infection**
Infective endocarditis, Tuberculosis, HIV, Amoeba infection
**Malignancies**
Hematological neoplasia
**Drugs**
Hydralazine, Propylthiouracil, Levamisole, Minocycline, Cocaine.
^#^The box is based upon previous reviews on related topics (6, 27). Serum ANCAs should be tested according to the algorithm proposed by the 2017 consensus on testing of ANCAs (6).	^*^IgA-vasculitis can show positive IgA-ANCAs in the active stage of disease, but IgG-ANCAs were rarely reported (27).	AAV, anti-neutrophil cytoplasmic antibody-associated vasculitis; ANCA, anti-neutrophil cytoplasmic antibodies; GBM, glomerular basement membrane; HIV, human immunodeficiency virus.

Differential diagnosis can be further narrowed down to entities with both similar CNS manifestations and ANCA positivity. These mainly include systemic inflammatory diseases (IgG4 related-disease, rheumatoid arthritis, systemic lupus erythematosus, Bechet syndrome), infective diseases (tuberculosis, human immunodeficiency virus) and malignancies (lymphoma).

In particular, diagnosis of AAV can be especially difficult at an early stage with only CNS presentations and a seronegative ANCA test, which may be an under-recognized common occurrence (24). Characteristics and diagnostic criteria of this entity remain to be elucidated. Differential diagnosis is therefore protean, varying according to the specific CNS manifestation of the patient. We suggest a thorough investigation of systemic involvement to exclude alternative causes including infection and malignancy. In particular, A continued monitoring of ANCA testing may be useful for those with idiopathic hypertrophic pachymeningitis (24). Most importantly, biopsy of the lesion is highly recommended in such cases to confirm the diagnosis.

## Treatment

**Figure 2:**
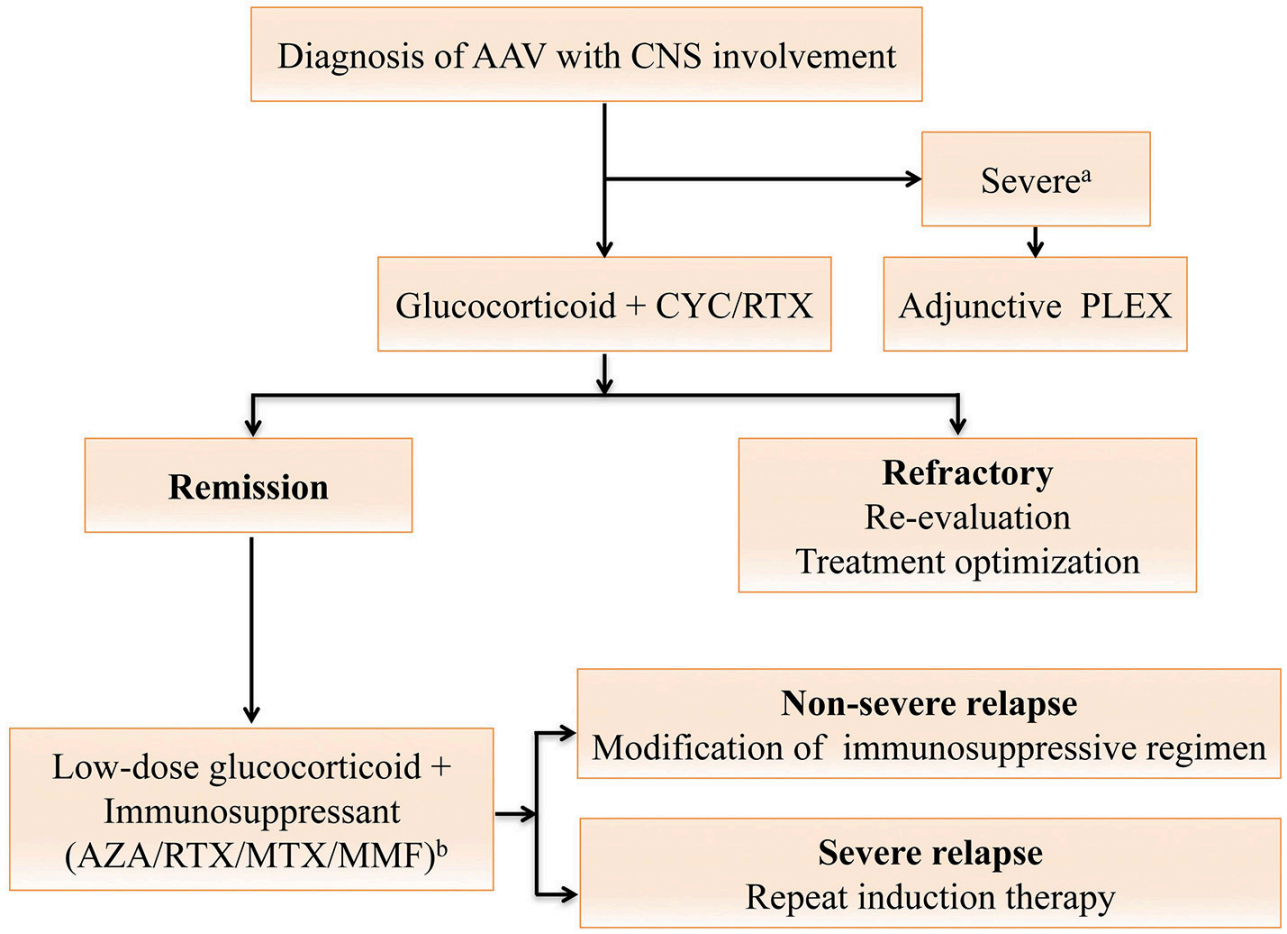
Treatment algorithm for anti-neutrophil cytoplasmic antibody (ANCA)-associated vasculitides (AAV) with central nervous system (CNS) involvement. The algorithm is formulated and terms are defined according to the 2016 European league Against Rheumatism (EULAR)/European Renal Association-European Dialysis and Transplant Association (ERA-EDTA) recommendations (61). For AAV with CNS involvement, the remission-induction therapy mainly consists of high-dose steroids and cyclophosphamide (CYC), or rituximab (RTX) for CYC-intolerant patients. Plasma exchange should be considered for those with a serum creatine level of ≥500 μmol/L or diffuse alveolar hemorrhage. Once complete remission is achieved, patients should be switched to the maintenance regimen. A combination of low-dose steroid and an oral immunosuppressive agent including azathioprine (AZA), methotrexate (MTX), mycophenolate mofetil (MMF) or RTX is used for at least 24 months (61). For patients refractory to the remission-induction therapy, referral to experts for reevaluation and treatment optimization is warranted (61). Severe relapses with organ- or life-threatening conditions are treated as per new disease, while non-severe relapses are managed with modification of the previous immunosuppressive regimen (61). ^a^AAV with severe renal impairment or diffuse alveolar hemorrhage; ^b^Drugs are listed in order of the strength of vote. AAV, anti-neutrophil cytoplasmic antibody-associated vasculitides; ANCA, anti-neutrophil cytoplasmic antibodies; AZA, azathioprine; CNS, central nervous system; CYC, cyclophosphamide; MTX, methotrexate; MMF, mycophenolate mofetil; PLEX, plasma exchange; RTX, rituximab.

Treatment should be started in a timely manner for patients highly suspected of AAV, even in the absence of pathological evidence. Steroid is an essential part of therapy, with the addition of a well-chosen immunosuppressant critical to prevent relapses and achieve remission of CNS symptoms in the long run. The treatment algorithm is shown in Figure 2. There are two phases of treatment, namely remission-induction and remission-maintenance. The choice of regimen in each phase depends mainly on the disease stage of the patient, as per the European Renal Association—European Dialysis and Transplant Association (ERA-EDTA) management recommendations for AAV (61). Generally, CNS involvement is regarded as an organ-threatening manifestation in AAV, especially for those with meningeal inflammation or retro-orbital disease. Remission-induction treatment for organ-threatening AAV typically consists of high-dose glucocorticoids and oral or intravenous (IV) cyclophosphamide (CYC) (1), termed CYC-based therapy. Glucocorticoids usually start with the dose of 1g intravenous methylprednisolone on 3 consecutive days, followed with 1 mg/kg daily oral corticosteroids (up to 80 mg per day), and a gradual reduction to daily dose of 7.5 to 10 mg within 3–5 months (7, 10). CYC can be given either intravenously in pulses or orally. The intravenous CYC, at a dose of 15 mg/kg (up to 1200 mg), are prescribed every 2 weeks initially and every 3 weeks from the 4th pulse. Daily oral CYC is given at a dose of 2 mg/kg/d. The CYC-based therapy is effective in 70-90% of patients (70), and the oral regimen can better prevent relapses (71). However, the daily oral regimen, compared with intravenous CYC, also poses more safety issues due to the cumulative toxicity (71, 72) including infertility, bladder hemorrhage, severe cytopenias, serious infection, and an increased risk of malignancy. Apart from the most recognized CYC-based therapy, the identification of the important role of B cells in the pathogenesis of AAV facilitated the use of rituximab (RTX) as an alternative to CYC (73). Two large randomized trials revealed RTX (375 mg/m^2^, once a week for four infusions) to be equivalent to CYC in terms of efficacy and safety in remission induction for AAV among treatment-naïve patients, and likely superior for relapsing disease (74).

Once complete remission (absence of any disease activity, usually attained after 8–12 weeks of treatment) is achieved, patients should be started with maintenance therapy to prevent further relapses, which should be continued for at least 24 months (61). In the remission maintenance phase, the induction regimen is switched to a combination of low-dose glucocorticoids and an oral immunosuppressive agent such as azathioprine (AZA), methotrexate (MTX), mycophenolate mofetil (MMF), or RTX (61). AZA (2 mg/kg/day) is the most commonly used immunosuppressive agent for remission maintenance, with MTX (20–25 mg/kg/week) as a similar alternative (72). AZA, with a lower relapse rate, is generally preferred over MMF. However, the relapse rate remains as high as 40% by 2 years despite the maintenance therapy with AZA or MTX (75). RTX is a potentially safer maintenance drug with a higher efficacy (76). However, the utility and toxicity profile of RTX require further confirmation (76–80). In addition, the adjunctive use of plasma exchange (PLEX) (7 sessions over 2 weeks) in AAV patients with severe renal dysfunction and/or alveolar hemorrhage seems reasonable in the short-term, but remains elusive in the long run (81).

Another issue to tackle in AAV is the treatment response. Refractory to remission-induction treatment is defined as follows: (1) unchanged or increased disease activity after 4 weeks of treatment (2) < 50% reduction in the disease score [e.g., Birmingham Vasculitis Activity Score (BVAS)] after 6 weeks of treatment (3) Presence of at least one major or three minor items on the disease activity score after over 12 weeks of treatment (61, 82). For AAV patients refractory to therapy, reevaluation and optimization of treatment, in close collaboration with experts, are recommended (61). Adjunctive intravenous immunoglobulin may help for those with persistent low disease activity (61, 83). Furthermore, relapses are not uncommon in AAV. Major relapses with organ- or life-threatening conditions are treated as per new disease, while non-severe relapses should be managed with an escalation or modification of the previous immunosuppressive regimen (61).

## Conclusion and Future Perspectives

For neurologists, prompt diagnosis of AAV in patients with CNS presentations allows timely treatment and thus a dramatic improvement in prognosis. A myriad of CNS presentations including hypertrophic pachymeningitis, ischemic stroke, intracranial hemorrhage and pituitary dysfunction, combined with certain systemic symptoms, raise the suspicion of AAV. Further ancillary tests are required, among which ANCA testing yields a great diagnostic value. ANCA positivity strongly suggests the diagnosis of AAV but ANCA negativity does not rule out the diagnosis. Once diagnosed, early treatment with steroid and immunosuppressant is essential to prevent neurological relapses and sequelae.

Recent evidence suggests the presence of “CNS-limited AAV” as a distinct subset in AAV. Efforts to better elucidate its phenotypic features, optimal treatment and long-term outcome are an important focus of future research. Furthermore, accumulating studies suggest that PR3-ANCAs and MPO-ANCAs define distinctive conditions among patients with AAV. Whether the same rule applies to the neurological conditions in CNS-involved AAV, however, remains unknown. Continued attempts are needed to validate the utility of ANCA specificity in classifying CNS manifestations, guiding treatment decisions, and predicting prognosis.

## Author Contributions

YZ: contributed to data collection, conception of the work and drafting the manuscript. YxZ: conception of the work and revising the manuscript. MC: revising the manuscript and figures. NL: revising the manuscript and tables. ZC and MD: revising the manuscript and final approval of the version to be published.

### Conflict of Interest Statement

The authors declare that the research was conducted in the absence of any commercial or financial relationships that could be construed as a potential conflict of interest.
